# Inter-subject correlations and their behavioral associations vary across movies: Implications for generalizability

**DOI:** 10.1162/IMAG.a.1400

**Published:** 2026-10-16

**Authors:** Simon Leipold, Rajat Ravi Rao, Jan-Mathijs Schoffelen, Sara Bögels, Ivan Toni

**Affiliations:** Centre for Cognitive Neuroimaging, Donders Institute for Brain, Cognition, and Behaviour, Radboud University, Nijmegen, The Netherlands; Social Brain Sciences Lab, Department of Humanities, Social and Political Sciences, ETH Zurich, Zürich, Switzerland; Neuroscience Center Zurich, University of Zurich and ETH Zurich, Zürich, Switzerland; Cognitive Psychology, Leiden University, Leiden, The Netherlands; Centre for Language Studies, Radboud University, Nijmegen, The Netherlands

**Keywords:** naturalistic neuroimaging, neural synchronization, movie-watching fMRI, individual differences, IS-RSA

## Abstract

Movie-watching functional magnetic resonance imaging (fMRI) has become increasingly popular in neuroscience. Movie-fMRI data are commonly analyzed using inter-subject correlation (ISC), which quantifies the similarity of neural time series across individuals. Differences in ISC during movie viewing have been associated with psychological traits and clinical diagnoses. However, most studies investigating group differences in ISC or ISC–behavior associations have drawn conclusions from a single movie. Because ISC is inherently stimulus driven, effects observed for one movie may not generalize to another. Yet, the extent to which ISC patterns and ISC–behavior associations depend on the specific movie being viewed has received limited systematic attention. Here, we analyzed three independent datasets comprising 318 subjects and 36 movies in total to quantify between-movie variability in ISC and assess its consequences for ISC–behavior associations. Across datasets, ISC varied between movies throughout the cortex. This variability was spatially heterogeneous: regions with stronger ISC showed greater between-movie variability. Movie-specific inter-subject representational similarity analysis revealed distinct spatial distributions of ISC–behavior associations, with little overlap between movies. This pattern was observed for two distinct behavioral constructs. These findings suggest that ISC–behavior associations can be strongly movie specific.

## 1 Introduction

Over the past two decades, movie-watching paradigms have become increasingly popular in neuroscience (Eickhoff et al., 2020; Hasson et al., 2010; Jääskeläinen et al., 2021; Redcay & Moraczewski, 2020; Sonkusare et al., 2019; Vanderwal et al., 2019). In these paradigms, subjects view movies while undergoing neuroimaging, most commonly functional magnetic resonance imaging (fMRI). A common approach for analyzing fMRI data acquired during movie watching is inter-subject correlation (ISC) analysis (Hasson et al., 2004). In ISC analysis, the fMRI time series from a given brain region, such as a voxel or atlas parcel, in one subject is used as a model for the fMRI time series from the same region in another subject (Nastase et al., 2019). The resulting correlation coefficient quantifies the similarity, or synchronization, of neural activity between subjects (Nummenmaa et al., 2018). ISC can be computed pairwise across all possible subject pairs or by correlating each subject’s time series with the group-average time series. The resulting correlations are then typically summarized, for example by averaging across subject pairs, to estimate the overall level of synchronization within each brain region. These group-level estimates are themselves commonly referred to as ISC.

Prior work has demonstrated that movies evoke strong ISC across the cortex, with particularly strong synchronization in visual and auditory brain regions (Hasson et al., 2004; Nummenmaa et al., 2018). An advantage of ISC over general linear model analysis is that it is data driven and does not require explicit regressors for specific event types, such as the appearance of faces or objects during the movie. Crucially, ISC is *stimulus driven*: activation changes that are not consistently related to processing of the movies across subjects remain largely uncorrelated and do not contribute to ISC estimates (Nastase et al., 2019; Nummenmaa et al., 2018). This stimulus sensitivity is a key strength of ISC, as it allows researchers to identify brain responses that are reliably driven by shared stimulus processing. However, the extent to which different movies evoke similar or distinct spatial patterns of ISC has rarely been examined systematically.

In recent years, studies using movie-fMRI paradigms have increasingly examined group differences in ISC. In a typical study, ISC is compared between groups or subgroups of subjects defined by characteristics such as psychological traits or clinical diagnoses. For example, friends show higher ISC than individuals who are more distant from one another in a social network (Parkinson et al., 2018). Similarly, sisters show higher ISC than friends (Bacha-Trams et al., 2024), and married couples show higher ISC than randomly paired individuals (L. Li et al., 2022). In the clinical domain, studies have reported altered ISC during movie watching in individuals with autism (Hasson et al., 2009; Mangnus et al., 2025; Salmi et al., 2013), psychosis (Mäntylä et al., 2018), depression (Guo et al., 2015), and social anxiety (Camacho et al., 2024; Koch et al., 2026), among other clinical populations. This line of research has recently been extended through inter-subject representational similarity analysis (IS-RSA), which enables a dimensional assessment of the association between subjects’ neural synchronization patterns and behavioral characteristics (Finn et al., 2020; Nummenmaa et al., 2012). For example, subjects who are more similar in personality traits (Matz et al., 2022) or political orientation (de Bruin et al., 2023; Katabi et al., 2023; Leong et al., 2020; van Baar et al., 2021) show higher ISC during movie watching than subjects who are less similar in these characteristics.

Most studies investigating group differences in ISC or associations between ISC and behavior have drawn conclusions from a single film or movie clip. However, the few studies using multiple movies suggest that different movies can evoke distinct spatial patterns of neural synchronization across the brain (Camacho et al., 2024; Katabi et al., 2023; L. Li et al., 2022; Sievers et al., 2024; van Baar et al., 2021). This is plausible because movies are spatially and temporally complex, highly multidimensional stimulus sequences that vary across numerous perceptual, narrative, affective, and stylistic features (Bartels & Zeki, 2004; Grall & Finn, 2022; Hasson et al., 2004; Schmälzle & Huskey, 2023). Indeed, the sensitivity of ISC to such features was already highlighted in the earliest ISC studies (Hasson et al., 2004, 2008, 2010). Because ISC is inherently stimulus driven (Nastase et al., 2019; Nummenmaa et al., 2018), group differences or ISC–behavior associations observed for one movie may not necessarily generalize to another. However, the extent and interpretational consequences of this movie specificity have received limited systematic attention.

The extent to which ISC findings generalize from one movie to another is central for defining the scope of conclusions drawn from studies using one or a few movies (Sonkusare et al., 2019; Vanderwal et al., 2019). We focus on generalizability across stimulus materials, defined here as the extent to which findings obtained with one movie would be expected to recur for other movies that could have been used to address the same research question (Yarkoni, 2022). This does not imply that findings from one movie should generalize to all possible movies. Rather, the question is how strongly ISC findings depend on the movie selected, and whether they remain stable across other movies. Generalizability across movies is, therefore, a graded rather than all-or-none property. At one extreme, an ISC-based finding may generalize across many different movies; at the other, it may be specific to the particular movie used in a study.

Here, we leverage 3 independent datasets, comprising 1 discovery dataset and 2 replication datasets, with 36 movies and 318 subjects in total, to investigate how ISC and ISC–behavior associations vary across movies (Fig. 1). In the discovery dataset (*CABB*), 112 adults each watched 8 animated short films (Eijk et al., 2022) and completed a task assessing conceptual judgments of novel objects, which we related to ISC using IS-RSA. In the first replication dataset (*HCP*), 178 subjects watched 14 short live-action movie clips (Van Essen et al., 2013); in the second replication dataset (*Emo-FilM*), 28 subjects watched 14 live-action and animated short films (Morgenroth et al., 2025). For both replication datasets, we related similarity between subjects in Big Five personality traits (Goldberg, 1993) to ISC. Across the three datasets, ISC varied between movies across the cortex, with the extent of between-movie variability differing across brain regions: regions with stronger ISC showed greater variability. Movie-specific IS-RSA revealed strongly varying ISC–behavior associations across movies, a pattern observed for both behavioral constructs.

**Fig. 1.**
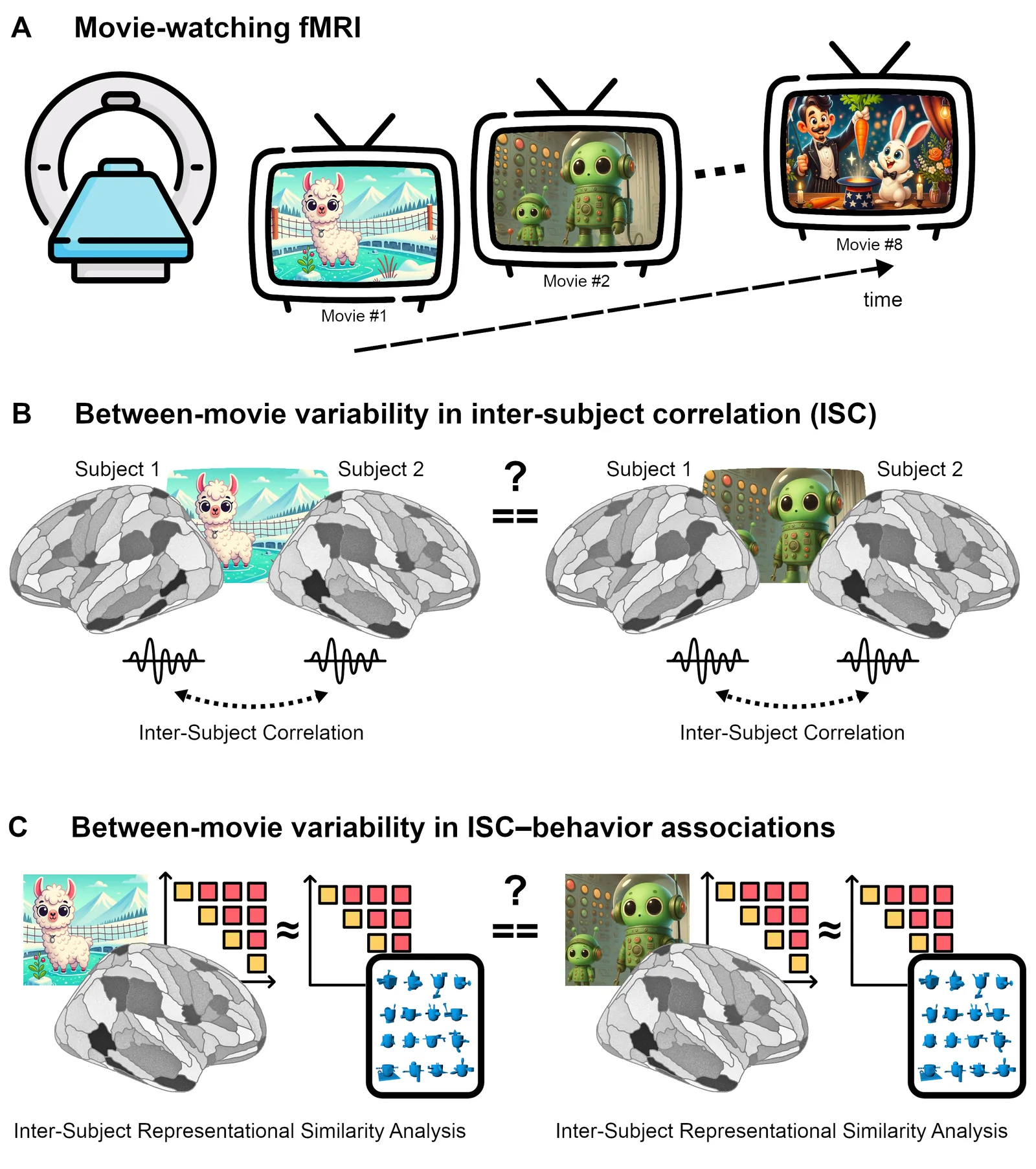
Overview. (A) In the discovery dataset (CABB), subjects (*N* = 112) viewed eight animated movies during fMRI scanning. The movies were selected to include diverse object categories, such as humans, plants, tools, toys, and food. (B) We quantified the variability in inter-subject correlation (ISC) between movies across the brain. For each subject and movie, we extracted a spatially averaged fMRI time series from 210 cortical parcels. ISC was computed for each parcel by correlating the time series between all subject pairs. (C) To assess the consequences of between-movie variability for ISC–behavior associations, we used inter-subject representational similarity analysis (IS-RSA) to relate neural synchronization during movie watching to the similarity of subjects’ conceptual representations of novel objects (“Fribbles”, in blue), with analyses conducted separately for each movie.

## 2 Methods

### 2.1 Discovery dataset: Communicative Alignment of Brain and Behavior (CABB)

#### 2.1.1 Subjects

We analyzed data from *N* = 112 subjects from the Communicative Alignment of Brain and Behavior (CABB) dataset (Eijk et al., 2022). These subjects were the same as those included in the fMRI analysis in the dataset description paper by Eijk et al. (2022) and did not include subjects who were excluded due to technical malfunctions, incomplete data, or poor data quality. Subjects had a mean age of 22.72 years (standard deviation = 3.21 years); 36 were male and 76 were female. All subjects reported no history of neurological or language-related disorders, no brain surgery, no hearing impairments, no metal implants other than dental implants, and normal or corrected-to-normal vision. Written informed consent was obtained from all subjects for being included in the study. For our main analysis, a repeated-measures analysis of variance (ANOVA) on ISC values of 56 subject pairs, we had 80% statistical power to detect differences in ISC between movies with an effect size of Cohen’s *f* > 0.51. Statistical power was calculated using the *WebPower* package (version 0.9.2) (Zhang & Yuan, 2018) in *R* (version 4.1.0; RRID:SCR_001905).

#### 2.1.2 fMRI movie tasks

Subjects viewed eight animated movies (see Supplementary Methods for details), which were presented using Presentation software (version 20.2; RRID:SCR_002521). Each movie was a complete animated short film with a self-contained narrative, rather than an excerpt from a longer film. The movies were selected to include categories of objects frequently mentioned in pilot tests of novel objects (“Fribbles”, Fig. 1C) used as referents by the subjects during face-to-face dialogues, such as humans, plants, tools, toys, and food. These animated movies are not geared exclusively toward children, but are accessible and engaging for a general audience, including adults. The movies were presented on a portion of the screen slightly below the center, on a black background with a height of 360 pixels and a width of 640 pixels, with a visual angle of approximately 9–11° vertically and 16–20° horizontally. The movies were played at 30 frames per second. Each movie was played in its entirety, except for the start and end (i.e., titles and credits), which were cut off to ensure no text was shown to subjects. The average duration of the movies was 4.1 minutes (range = 2.2–6.1 minutes), totaling 35 minutes, including 12-second breaks between subsequent movies. The movies were preceded by a filler video clip that lasted a few seconds. Subjects were instructed to simply attend to the movies and to lie still. The movies did not contain spoken language, but the original soundtrack was provided to subjects via earphones. Before scanning began, the sound levels were adjusted to each subject’s individual preferences.

#### 2.1.3 Imaging data acquisition

Magnetic resonance images were acquired using two 3T MAGNETOM magnetic resonance imaging scanners: Prisma and PrismaFit (Siemens AG, Healthcare Sector, Erlangen, Germany). Blood oxygenation level dependent (BOLD) functional images were acquired using a multi-band 2D-echo planar imaging (EPI) sequence (Uğurbil et al., 2013) with repetition time (TR) = 1000 ms, echo time (TE) = 34 ms, flip angle = 60º, 2 mm isotropic resolution, field of view (FOV) = 208 x 208 mm. A multi-band acceleration factor of 6 was used in the slice direction, and no parallel imaging was applied in plane. Phase encoding was applied in the anterior–posterior (AP) direction with a partial Fourier coverage of 7/8. About 2074 volumes were acquired during movie watching. Anatomical images were acquired with a T1-weighted 3D MPRAGE sequence, integrated parallel acquisition technique (iPAT) acceleration factor = 2, with TR = 2200 ms, TE = 2 ms, inversion time (TI) = 1100 ms, flip angle = 11°, 0.8 mm isotropic resolution, and FOV = 256 x 240 x 166 mm; with a T2-weighted turbo spin echo (TSE) sequence with variable flip angle, TR = 3200 ms, TE = 569 ms, echo spacing = 4.12 ms, turbo factor = 314, 0.8 mm isotropic resolution, and FOV = 256 x 240 x 166 mm. Raw imaging data were converted to Brain Imaging Data Structure (BIDS) format (Gorgolewski et al., 2016) using *BIDScoin* (version 1.5; RRID:SCR_022839) (Zwiers et al., 2022).

#### 2.1.4 Imaging data preprocessing

Each subject’s raw imaging data underwent a quality control assessment for excessive motion, susceptibility artifacts, or other sources of noise using *MRIQC* (version 23.1.0; RRID:SCR_022942). Imaging data preprocessing was implemented using *fMRIPrep* (version 20.2.7; RRID:SCR_016216) (Esteban et al., 2019), which is based on *Nipype* (version 1.7.0; RRID:SCR_002502) (Gorgolewski et al., 2011). The main preprocessing steps included segmentation, surface reconstruction, and spatial normalization of the T1-weighted anatomical image; and slice timing correction, susceptibility distortion correction, head-motion correction, and spatial normalization of the fMRI data. Details of the preprocessing pipeline are included in the Supplementary Methods.

#### 2.1.5 fMRI data denoising

Each subject’s preprocessed fMRI data underwent further denoising, implemented using *Nilearn* (version 0.9.2; RRID:SCR_001362) in *Python* (version 3.7.13; RRID:SCR_008394). The voxel-wise fMRI time series were detrended and low-pass filtered at 0.08 Hz. Next, the following confounds estimated by fMRIprep during preprocessing were regressed out from the voxel-wise time series: six motion parameters (three translations, three rotations) and their first temporal derivatives, as well as cerebrospinal fluid and white matter time series and their first temporal derivatives. In addition, scans that exceeded a threshold of 0.5 mm framewise displacement (FD) or 1.5 standardized DVARS were annotated as motion outliers and included as nuisance regressors. Subsequently, the time series were standardized to have a mean of zero and unit variance. After denoising, we segmented the fMRI time series and extracted a separate time series for each movie based on the onset and offset times recorded in the Presentation log files. The denoised fMRI data for each movie were then spatially smoothed with a 6 mm full-width-at-half-maximum (FWHM) three-dimensional Gaussian kernel.

#### 2.1.6 Inter-subject correlation (ISC) analysis

To calculate parcel-wise ISC values, we first extracted a spatially averaged time series for each subject and movie from the cortical parcels of the Brainnetome atlas (Fan et al., 2016) using *NLtools* (version 0.4.7) (Chang et al., 2022) in *Python*. We restricted the analyses to the 210 cortical parcels of the Brainnetome atlas, excluding the 36 subcortical parcels, because the IS-RSA focused on conceptual representations of novel objects, which we expected to be primarily reflected in cortical synchronization patterns. For each movie and cortical parcel, we then calculated the ISC values for all possible subject pairs by computing the Pearson correlation coefficient between the parcel-wise fMRI time series of subject [i] and subject [j] of each pair [i,j] using *scikit-learn* (version 1.0.2; RRID:SCR_002577) in *Python*. To obtain a mean ISC value for each parcel, we averaged the ISC values across all subject pairs. Visualization of the ISC values in the brain was implemented using *Nilearn*.

#### 2.1.7 Statistical analyses of ISC values

Prior to statistical analysis, all ISC values were transformed using Fisher’s r-to-z transformation to normalize the distribution of correlation coefficients. To quantify between-movie variability in ISC across the whole cortex, we averaged the ISC values across all cortical parcels to obtain a single ISC value per subject pair and movie. Then, we entered the whole-brain ISC values into a repeated-measures ANOVA with Movie as the within-subject factor. We used data from 56 subject pairs, ensuring that each subject contributed only to a single ISC value per movie and that the assumptions of the repeated-measures ANOVA were not violated. Next, we repeated the ANOVA for each parcel separately to obtain a spatial distribution of between-movie variability across the cortex. The ANOVAs were performed using the *ez* (version 4.4-0; RRID:SCR_020990) and *rstatix* (version 0.7.2; RRID:SCR_021240) packages in *R*.

To investigate whether brain regions with high levels of ISC also show high variability in ISC, we correlated the parcel-wise level of ISC with the parcel-wise between-movie variability in ISC. Specifically, we extracted the *F* values from the repeated-measures ANOVAs for each parcel as a measure of between-movie variability. We then calculated the Pearson correlation coefficient between the *F* values and the ISC values for each parcel in *R*.

#### 2.1.8 Inter-subject representational similarity analysis (IS-RSA)

The CABB dataset encompasses behavioral data on subjects’ conceptual representations of novel objects (“Fribbles”, Fig. 1C), which were estimated in a *Features task* completed before the movie-fMRI tasks (Binder et al., 2016; Eijk et al., 2022). In this task, subjects used a linear visual analog scale to report how well each novel object matched 29 different features (e.g., pointy, symmetrical, human, related to movement; full list in Supplementary Table S1; see Supplementary Methods for details on the Features task). Subjects showed substantial individual differences in these feature ratings, indicating variability in how the novel objects were conceptually represented across individuals (Supplementary Fig. S1). Prior work has shown that subjects with similar interpretations of a narrative show higher neural synchronization than subjects with different interpretations (Yeshurun et al., 2017). Furthermore, subjects who share their interpretations of an ambiguous animation of geometric shapes show higher neural synchronization than those with dissimilar interpretations (Nguyen et al., 2019). Inspired by these previous studies, we hypothesized that subjects with similar conceptual representations of novel objects would show higher ISC during movie watching. As these particular movies were selected to contain categories of objects that were mentioned often in pilot tests to be associated with the novel objects (Eijk et al., 2022), we anticipated a “spill-over” effect from representations of novel objects to naturalistic viewing of conventional objects.

We conducted a regression-based IS-RSA (Finn et al., 2020; Nummenmaa et al., 2012) using linear mixed-effects models (G. Chen et al., 2017; van Baar et al., 2021) to link subject similarity in conceptual representations of the novel objects, estimated in the Features task, with neural synchronization during movie watching, separately for each movie. The linear mixed-effects models were estimated using the *lme4* package (version 1.1-33; RRID:SCR_015654) in *R* (version 4.1.0; RRID:SCR_001905). We estimated a full model to simultaneously assess the main effects of subjects’ similarities in conceptual representations on their ISC during movie watching, while controlling for subject similarity in lexical representations of the novel objects, estimated in a *Naming task*, and also controlling for subject similarities in sex and age. Details on the novel object stimuli, Features and Naming tasks, and the IS-RSA are given in the Supplementary Methods.

To ensure that our results were not driven by statistical thresholding, we additionally calculated Pearson correlations between all pairwise combinations of movie-specific, unthresholded IS-RSA *t*-maps and computed the average correlation separately for each dataset.

#### 2.1.9 Control analyses

We ran multiple additional analyses in the CABB dataset to test the robustness of our findings; these analyses are detailed in the Supplementary Results. Specifically, we (1) repeated all ISC and IS-RSA using the Schaefer 300-parcel atlas (Schaefer et al., 2018) instead of the Brainnetome atlas, to ensure that the results were not dependent on the specific anatomical parcellation scheme used in the main analyses; (2) repeated all ISC and IS-RSA using truncated time series to control for differences in movie length; (3) implemented a resampling approach to assess the stability of between-movie ISC variability across different non-overlapping subject-pair configurations, thereby testing whether the observed effects were robust to the particular pairing of subjects; (4) classified movies based on multivariate ISC patterns, to determine whether movie-specific ISC profiles were sufficiently distinct to identify individual movies from their spatial synchronization patterns; (5) controlled for differences in head motion across movies, to rule out the possibility that movie-specific differences in ISC were driven by systematic variation in subject motion; (6) controlled for parcel-wise temporal signal-to-noise ratio, to assess whether the association between overall ISC level and between-movie ISC variability could be explained by regional differences in data quality; (7) incorporated a random effect for movie in the IS-RSA model to evaluate whether any effects generalized across the movie set (van Baar et al., 2021); and (8) repeated the IS-RSA without covariates to confirm that the main findings were not dependent on the inclusion of control variables in the regression model.

### 2.2 Replication dataset 1: Human Connectome Project (HCP)

#### 2.2.1 Subjects

We analyzed data from the Human Connectome Project (HCP) S1200 release (Van Essen et al., 2013). After excluding 6 subjects with incomplete movie-watching data, the final sample included 178 subjects (mean age = 29.40 years, standard deviation = 3.31 years; 108 female, 70 male). The HCP study was approved by the Washington University Institutional Review Board, and all subjects provided informed consent.

#### 2.2.2 fMRI movie tasks

The HCP movie-watching protocol comprised two scanning sessions, each consisting of two runs of approximately 15 minutes. Each run included three to four short movie clips, separated by 20-second rest blocks, followed by a repeated validation clip. We excluded the validation clips because they consisted of concatenated brief clips lasting 1–3 seconds, resulting in 14 movie clips for analysis. Each analyzed clip was presented once. The analyzed clips had an average duration of 3.43 minutes, ranging from 1.06 to 4.30 minutes. Clips in the first and third runs were taken from independently produced Creative Commons films, whereas clips in the second and fourth runs were taken from Hollywood movies. Details on the movie clips are provided in the Supplementary Methods. All movie clips are available from the HCP website (https://db.humanconnectome.org/).

#### 2.2.3 Imaging data acquisition and preprocessing

Functional images were acquired on a 7T Siemens MAGNETOM scanner using a gradient-echo EPI sequence (Uğurbil et al., 2013) with TR = 1000 ms, TE = 22.2 ms, flip angle = 45º, 1.6 mm isotropic resolution, FOV = 208 x 208 mm, and a multiband acceleration factor of 5. We used the minimally preprocessed HCP data obtained from the HCP website. These data were processed with the standard HCP pipeline, including motion correction, spatial normalization, high-pass temporal filtering, regression of 24 motion parameters, and denoising (Glasser et al., 2013). We then segmented the preprocessed fMRI time series and extracted separate time series for each movie clip.

#### 2.2.4 ISC and IS-RSA

As in the CABB dataset, we computed parcel-wise ISC values by first extracting a spatially averaged time series for each subject and movie clip from the cortical parcels of the Brainnetome atlas, using *NLtools* (version 0.5.1) in *Python* (version 3.11.15). For each movie clip and cortical parcel, we then computed ISC values for all possible subject pairs as the Pearson correlation between the corresponding parcel-wise fMRI time series. All ISC values were Fisher transformed to normalize the distribution of correlation coefficients. Statistical analyses comparing ISC values across movie clips were identical to those used for the CABB dataset. For the repeated-measures ANOVA, we used data from 89 randomly sampled, non-overlapping subject pairs. We also correlated parcel-wise ISC levels with parcel-wise between-movie ISC variability, following the same procedure as in the CABB dataset.

The HCP dataset includes behavioral data on subjects’ Big Five personality traits, based on the well-established Five-Factor Model (Goldberg, 1993; Heine & Buchtel, 2009). Personality traits were assessed using the 60-item version of the Five-Factor Inventory, which has shown excellent reliability and validity (McCrae & Costa, 2004). To obtain the subject-by-subject matrix for IS-RSA, we computed the correlation distance between each pair of subjects based on their scores across the five personality traits: openness to experience (NEOFAC_O), agreeableness (NEOFAC_A), conscientiousness (NEOFAC_C), extraversion (NEOFAC_E), and neuroticism (NEOFAC_N). As in the CABB dataset, we conducted regression-based IS-RSA using linear mixed-effects models to link subject similarity in personality traits with neural synchronization during movie watching, separately for each movie clip. The linear mixed-effects models were estimated using the *lme4* package (version 1.1-35) in *R* (version 4.3.3).

### 2.3 Replication dataset 2: Emotion Research using Films and fMRI (Emo-FilM)

#### 2.3.1 Subjects

We analyzed the Emotion Research using Films and fMRI (Emo-FilM) dataset (Morgenroth et al., 2025). The Emo-FilM fMRI dataset included 30 healthy subjects. We excluded 2 subjects because of missing data, yielding a final sample of 28 subjects (mean age = 25.32 years, standard deviation = 3.44 years; 17 female, 11 male). All subjects were right handed, had normal or corrected-to-normal vision, full color vision, high English comprehension, no history of neurological or psychiatric conditions, and reported no use of neuropharmacological or recreational drugs. The study was approved by the Geneva Cantonal Commission for Ethics in Research (protocol No. 2018-02006), complied with the Code of Human Research Ethics (2014), and all subjects provided written informed consent.

#### 2.3.2 fMRI movie tasks

The Emo-FilM protocol comprised four fMRI sessions of approximately 2 hours each. Across these sessions, subjects watched 14 short films in pseudo-random order, with 2 to 5 films presented per session. The stimuli consisted of full live-action and animated short films with complete narratives and spoken language. The films were presented without beginning or end credits and had an average duration of 11.44 minutes, ranging from 6.70 to 17.14 minutes. Subjects were instructed to watch the films as they would in everyday life. Details on the films are provided in the Supplementary Methods.

#### 2.3.3 Imaging data acquisition and preprocessing

Functional images were acquired on a 3T Siemens MAGNETOM TIM Trio scanner using a 32-channel head coil and a gradient-echo EPI sequence: TR = 1300 ms, TE = 30 ms, flip angle = 64°, multiband acceleration factor = 3, 2.5 mm isotropic resolution, and FOV = 210 × 210 mm. We used preprocessed fMRI data downloaded from *OpenNeuro* (RRID:SCR_005031) via *DataLad* (RRID:SCR_003931) (Halchenko et al., 2021). These data were preprocessed separately for each movie. Preprocessing included motion correction, brain extraction, spatial normalization, spatial smoothing with a 6 mm FWHM Gaussian kernel, grand-mean intensity normalization, and high-pass temporal filtering. White matter and cerebrospinal fluid time courses were regressed out together with six motion parameters.

#### 2.3.4 ISC and IS-RSA

We computed parcel-wise ISC values by first extracting a spatially averaged time series for each subject and movie from the cortical parcels of the Brainnetome atlas. For each movie and cortical parcel, we then computed ISC values for all possible subject pairs as the Pearson correlation between the corresponding parcel-wise fMRI time series. All ISC values were transformed using Fisher’s r-to-z transformation. Statistical analyses comparing ISC values across movies were identical to those used for the CABB dataset. For the repeated-measures ANOVA, we used data from 14 randomly sampled, non-overlapping subject pairs. We also correlated parcel-wise ISC levels with parcel-wise between-movie ISC variability, following the same procedure as in the CABB dataset.

The Emo-FilM dataset includes behavioral data on subjects’ Big Five personality traits, based on the well-established Five-Factor Model (Goldberg, 1993; Heine & Buchtel, 2009). Personality traits were assessed using the Big Five Inventory (John et al., 1991). To construct the subject-by-subject matrix for IS-RSA, we computed the correlation distance between each pair of subjects based on their scores across the five personality traits: openness to experience (BIG5_ope), agreeableness (BIG5_agr), conscientiousness (BIG5_con), extraversion (BIG5_ext), and neuroticism (BIG5_neu). As in the CABB dataset, we conducted regression-based IS-RSA using linear mixed-effects models to link subject similarity in personality traits with neural synchronization during movie watching, separately for each movie. The linear mixed-effects models were estimated using the *lme4* package (version 1.1-35) in *R* (version 4.3.3).

## 3 Results

### 3.1 ISC patterns vary between movies

Our first aim was to quantify variability in ISC evoked by different movies across the cortex. We analyzed the discovery dataset (CABB), in which 112 subjects watched 8 animated short films.

As shown in Figure 2, the overall spatial distribution of ISC evoked by each of the eight movies resembled the patterns reported in previous movie-fMRI studies (Hasson et al., 2004, 2010; Vanderwal et al., 2019). The strongest ISC was observed in the extended visual system, including face-sensitive regions in the fusiform gyrus, and in the auditory system, including Heschl’s gyrus and the planum temporale. Strong ISC was also observed in higher-order temporal, parietal, and frontal cortices.

**Fig. 2.**
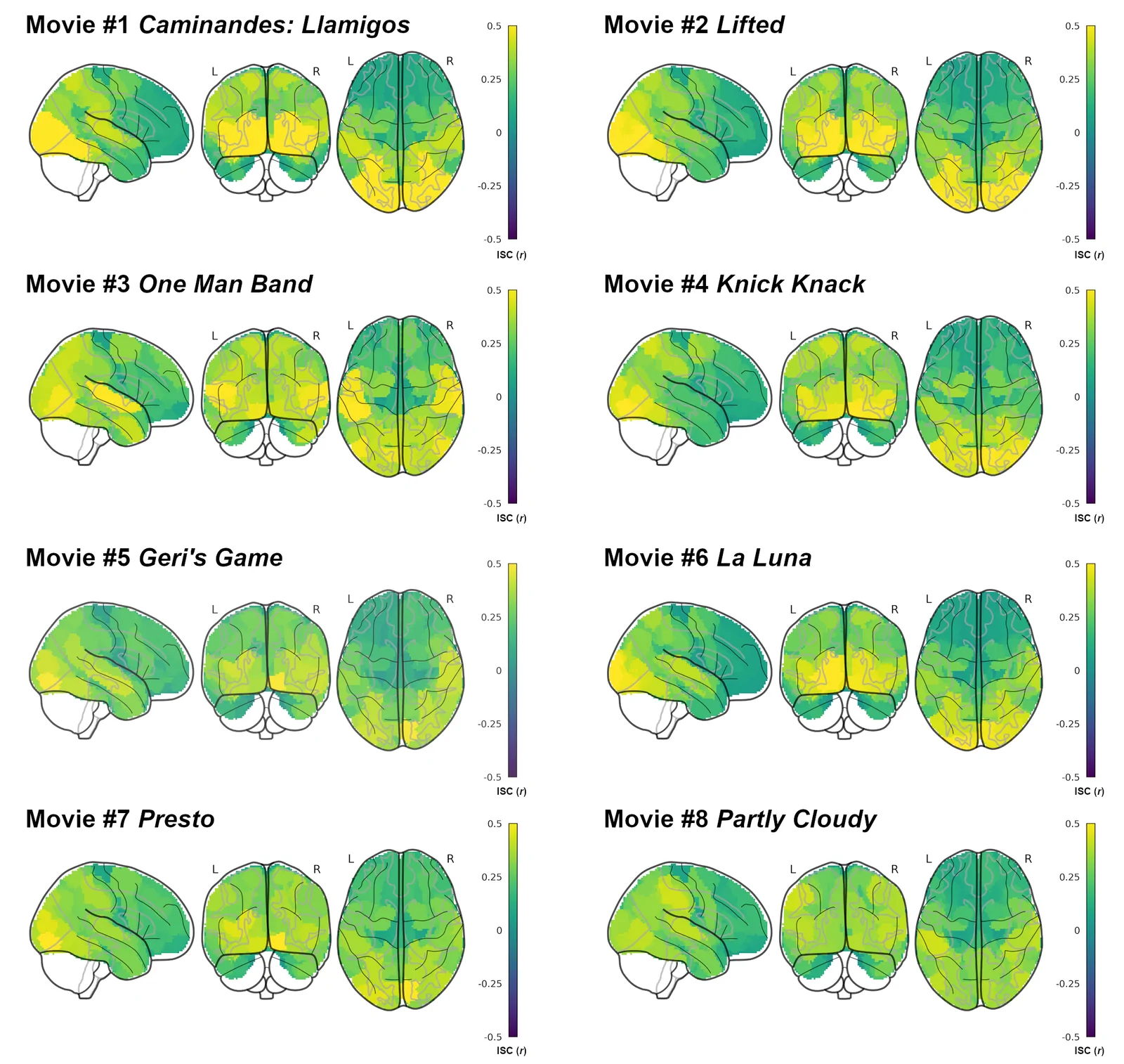
Inter-subject correlations for individual movies in the discovery dataset (CABB). Spatial distribution and magnitude of inter-subject correlations (ISC) across the cortex, shown separately for each of the eight movies.

We next quantified between-movie variability in ISC statistically. First, we tested whether whole-brain ISC varied between movies. To this end, we averaged ISC values across all cortical parcels to obtain one whole-brain ISC value per subject pair and movie. We then submitted the whole-brain ISC values to a repeated-measures ANOVA with Movie as the within-subject factor. To meet the assumptions of the ANOVA, we used a subset of non-overlapping subject pairs, ensuring that each subject contributed to only one pair. The ANOVA revealed a significant main effect of Movie (*F*(7, 385) = 4.93, *p* < 0.001, generalized eta-squared [η^2^_G_] = 0.051), indicating considerable variability in whole-brain ISC across movies. Figure 3A shows the mean and standard error of whole-brain ISC for each movie. The differences in ISC were not driven by a single movie differing from all others; rather, whole-brain ISC varied across the full set of movies.

**Fig. 3.**
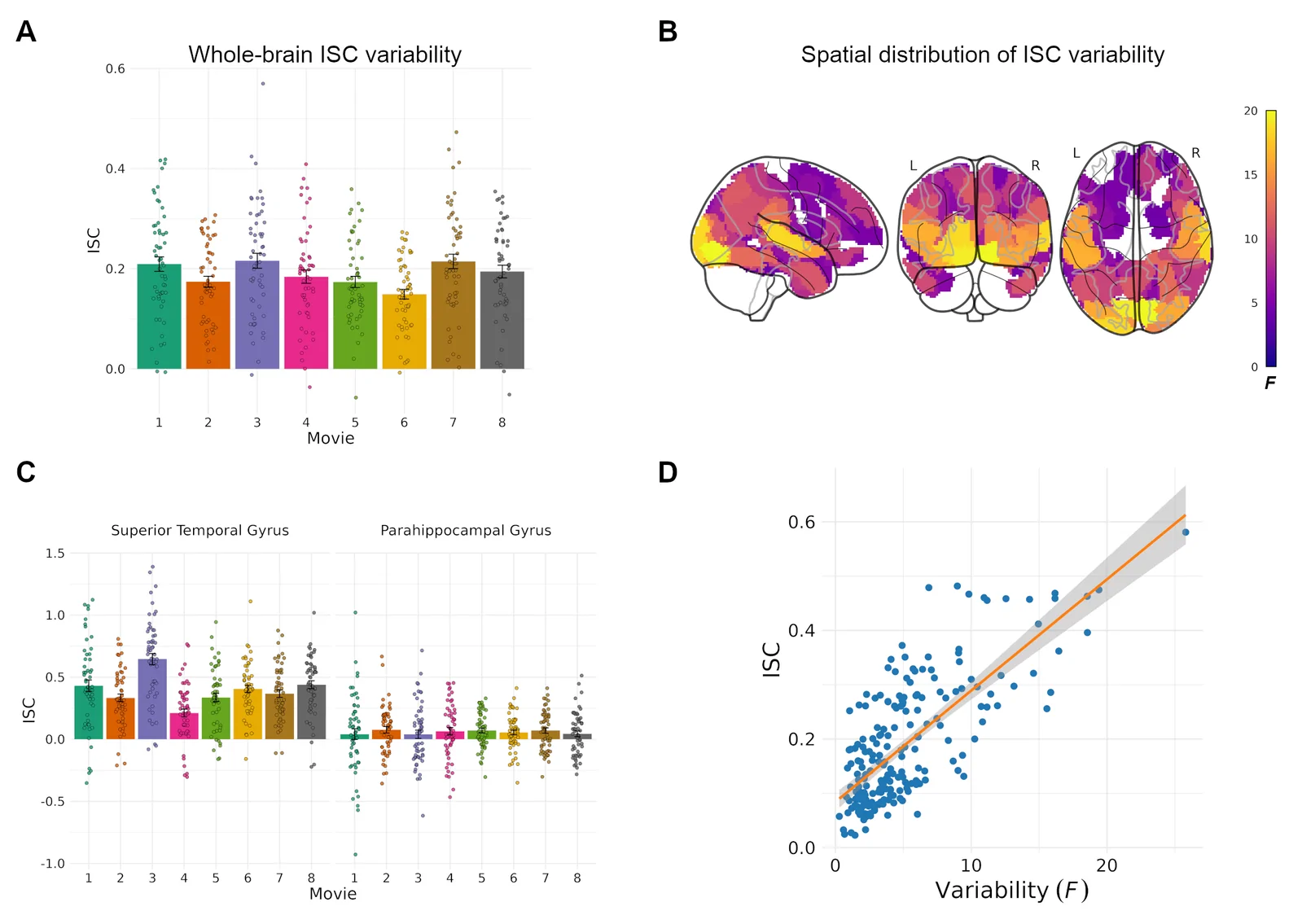
Variability in inter-subject correlations across movies in the discovery dataset (CABB). Fisher *z*-transformed ISC is shown for panels A, C, and D. (A) Mean and standard error of whole-brain inter-subject correlation (ISC) values for each of the eight movies. Whole-brain ISC was computed by averaging ISC values across all cortical parcels for each subject and movie. A repeated-measures ANOVA revealed a significant main effect of Movie, indicating considerable variability in whole-brain ISC across movies. (B) Spatial distribution of between-movie ISC variability across the brain. A significant main effect of Movie (family-wise error-adjusted *p* < 0.05) was found for 97 out of 210 cortical parcels (46.2%). The highest variability was observed in primary and associative visual cortices, as well as in primary and associative auditory cortices. Significant variability was also found in higher-order regions such as the precuneus and inferior parietal lobule. (C) Brain regions with high between-movie variability, such as the superior temporal gyrus, tended to show relatively higher ISC levels, while regions with low variability, such as the parahippocampal gyrus, showed lower ISC. (D) A strong positive correlation between ISC level (y-axis) and between-movie variability (x-axis) across brain regions indicates that regions with higher ISC levels also exhibit greater variability across movies. Each point represents one cortical parcel.

Next, we examined the spatial distribution of ISC variability across the cortex. For each of the 210 cortical parcels defined by the Brainnetome atlas, we conducted a repeated-measures ANOVA on ISC values with Movie as the within-subject factor. We found a statistically significant main effect of Movie in 97 of the 210 cortical parcels (46.2%; family-wise error [FWE]-adjusted *p* < 0.05). The strongest between-movie variability was observed in primary and associative visual cortex, as well as in primary and associative auditory cortex. Considerable variability was also observed in higher-order regions, including the precuneus and inferior parietal lobule. Parcel-wise statistical values quantifying between-movie variability are available online (https://dx.doi.org/10.17605/OSF.IO/S78WU). Figure 3B shows the spatial distribution of between-movie ISC variability across the cortex.

Importantly, these results were robust across additional control analyses. The pattern remained highly similar when using the Schaefer 300-parcel atlas (Schaefer et al., 2018) instead of the Brainnetome atlas (Supplementary Fig. S2), when using equal-length movie segments to ensure that ISC differences were not solely driven by differences in movie duration (Supplementary Fig. S4), and after residualizing ISC values for pairwise average FD, indicating that head motion did not explain between-movie ISC variability (Supplementary Fig. S6). Results also did not depend on the specific choice of subject pairs, as shown by a resampling analysis in which the ANOVA was repeated for 1000 randomly sampled sets of non-overlapping subject pairs (Supplementary Fig. S7).

To further test whether ISC patterns carry movie-specific information, we conducted a cross-validated multivariate classification analysis to distinguish movies based on parcel-wise ISC patterns. The classifier achieved an accuracy of 62.9%, substantially above chance level (12.5%; *p* = 0.001, permutation test). This indicates that the spatial distribution of ISC contains robust information about movie identity. The analysis is described in detail in the Supplementary Results and illustrated in Supplementary Figure S8.

### 3.2 Brain regions with stronger ISC show higher between-movie variability

We followed up on the finding of widespread between-movie ISC variability across the cortex by examining regions with particularly high or low variability. We discovered that brain regions showing high variability, such as the superior temporal cortex, also had relatively higher levels of ISC. Conversely, brain regions with low variability, such as the parahippocampal gyrus, had lower levels of ISC (Fig. 3C). To quantify this relationship, we calculated the Pearson correlation between parcel-wise ISC level and between-movie ISC variability across the 210 cortical parcels. As shown in Figure 3D, ISC level was strongly positively correlated with between-movie variability (*r* = 0.73, *t*(208) = 15.62, *p* < 0.001, *R*^2^ = 0.54). This association remained strong after controlling for parcel-wise temporal signal-to-noise ratio calculated from preprocessed time series, partial *r* = 0.73, *t*(207) = 15.45, *p* < 0.001, *R*^2^ = 0.54 (Supplementary Fig. S9).

### 3.3 ISC–behavior associations vary across movies

Our next objective was to assess the consequences of between-movie ISC variability for the generalizability of ISC–behavior associations. We used IS-RSA to identify brain regions in which similarity between subjects in conceptual representations of novel objects, as measured by the *Features task*, was associated with ISC during movie watching. In other words, we tested whether subjects with more similar conceptual representations of the novel objects showed stronger neural synchronization in specific brain regions.

For each movie separately, we performed regression-based IS-RSA using linear mixed-effects models (G. Chen et al., 2017) to estimate the association between subject similarity in conceptual representations and ISC during movie watching, while controlling for similarities in lexical representations, assessed with a *Naming task*, as well as age and sex. Linear mixed-effects models are commonly used to quantify ISC–behavior associations (Baek et al., 2022, 2023; de Bruin et al., 2023; Finn et al., 2018; Jangraw et al., 2023; Moraczewski et al., 2018; van Baar et al., 2021; Ye et al., 2026). If between-movie ISC variability had little impact on ISC–behavior associations, we would expect similar IS-RSA patterns across movies. By contrast, if between-movie variability is consequential for ISC–behavior associations, we would expect IS-RSA patterns to differ across movies, and in the strongest case, to be movie specific.

As shown in Figure 4, the IS-RSA revealed associations between conceptual representations of novel objects and ISC in distinct brain regions for each movie. For Movie #1, subject similarity in conceptual representations was significantly associated with neural synchronization in parcels within bilateral insula (right hemisphere: *p*_FWE_ = 0.004 and left hemisphere: *p*_FWE_ = 0.007), left inferior parietal lobule (*p*_FWE_ = 0.006), left precentral gyrus (*p*_FWE_ = 0.007), right superior frontal gyrus (*p*_FWE_ = 0.02), left inferior temporal gyrus (*p*_FWE_ = 0.03), and right anterior cingulate (*p*_FWE_ = 0.03). In contrast, for Movie #2, we found no statistically significant association between conceptual representations and neural synchronization. For Movie #3, significant associations were observed in the left precuneus (*p*_FWE_ < 0.05), with no overlap with the regions identified for Movie #1. For Movie #4, significant associations were observed in bilateral middle frontal gyrus (right hemisphere: *p*_FWE_ < 0.001 and left hemisphere: *p*_FWE_ = 0.03) and the right orbital gyrus (*p*_FWE_ = 0.02), again without overlap with the regions identified for Movie #1 or Movie #3. Similarly, Movies #5 through #8 showed largely non-overlapping associations between conceptual representations and neural synchronization, with the exception of one parcel in the right middle temporal gyrus that was significant for both Movie #5 (*p*_FWE_ = 0.004) and Movie #6 (*p*_FWE_ < 0.001). Statistically significant IS-RSA associations for all movies are detailed in Supplementary Table S2. Full parcel-wise results for all movies are available online (https://dx.doi.org/10.17605/OSF.IO/S78WU). Correlations between the movie-specific, unthresholded IS-RSA *t*-maps were low (mean *r* = 0.01 ± 0.13). This suggests limited consistency in the spatial patterns of ISC–behavior associations across movies, independent of the applied statistical threshold.

**Fig. 4.**
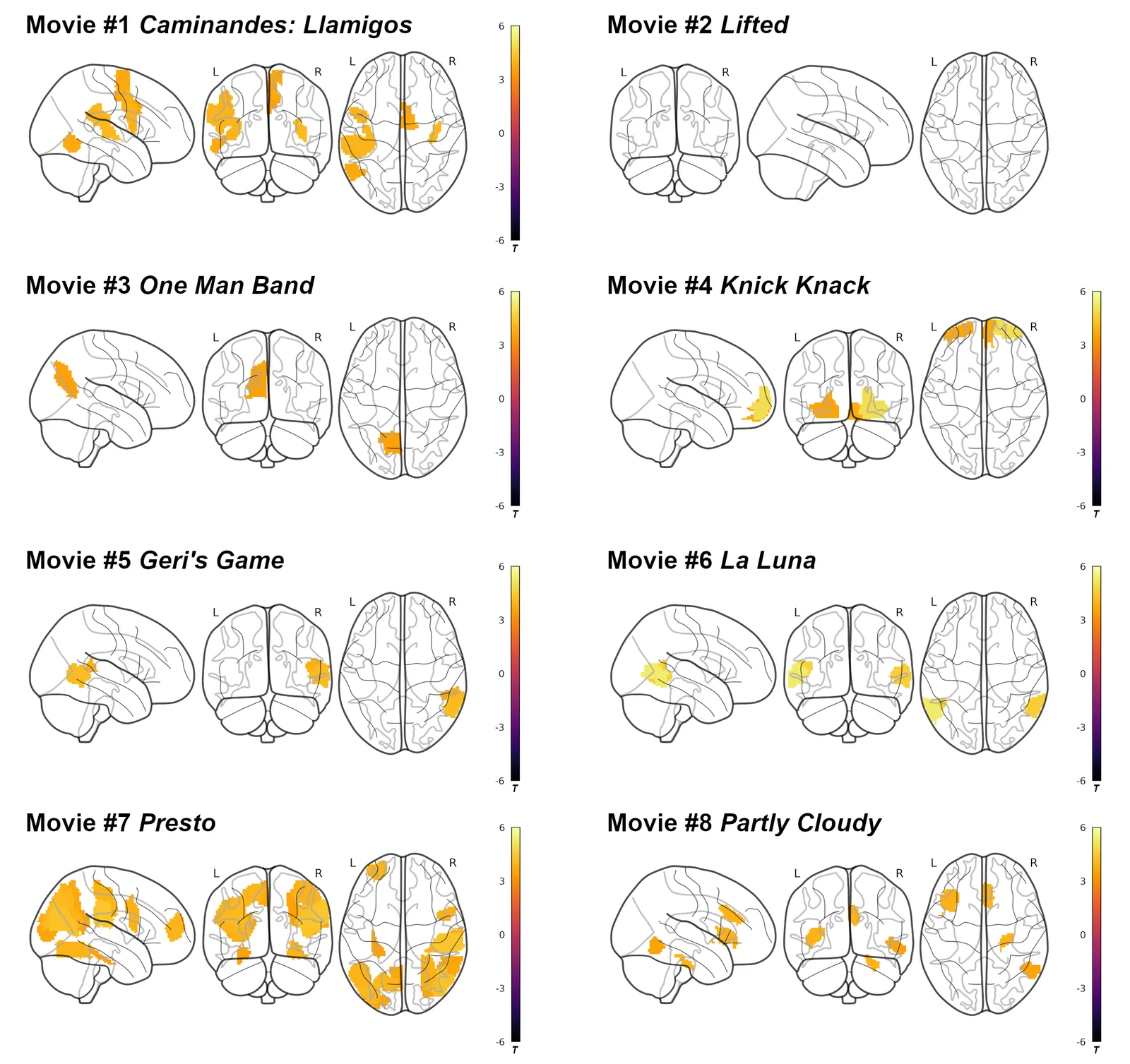
Between-movie ISC variability leads to varying IS-RSA associations between behavior and neural synchronization. We used inter-subject representational similarity analysis (IS-RSA) to identify brain regions where similarity in conceptual representations of novel objects was associated with similarity in neural synchronization during movie watching. The results revealed ISC–behavior associations in distinct sets of brain regions for each movie (family-wise error-adjusted *p* < 0.05).

A control analysis using the Schaefer 300-parcel atlas instead of the Brainnetome atlas yielded similar results (Supplementary Fig. S3). Because the higher number of parcels required stricter correction for multiple comparisons, fewer regions showed statistically significant IS-RSA effects, and Movie #8 showed no significant associations. In a further control analysis using equal-length movie segments, movie-specific IS-RSA patterns remained largely robust, although some effects changed after truncation (Supplementary Fig. S5). The disappearance of the Movies #6 and #7 IS-RSA effects after truncating the time series further suggests that ISC–behavior associations may depend not only on the movie, but also on which segment of the movie is analyzed. Finally, a control analysis without covariates yielded highly similar IS-RSA results, indicating that the findings were robust and not driven by overlap with lexical representations or demographic similarity (Supplementary Fig. S10).

### 3.4 A subset of IS-RSA effects generalize across movies

To examine whether any associations in the discovery dataset (CABB) generalized across movies, we conducted an additional IS-RSA that included movie as a random effect. This analysis identified three brain regions, including bilateral posterior inferior temporal gyrus and right posterior superior temporal sulcus, where ISC was significantly associated with conceptual representations of novel objects across movies (Supplementary Fig. S11 and Supplementary Table S3). Because this model pooled data across the eight movies, these effects are best interpreted as average associations across the sampled movies with increased statistical precision, rather than as evidence that the model removes movie-specific ISC–behavior effects.

### 3.5 Replication of the main findings in two independent datasets

To assess the robustness of our findings across different subjects, movie types, and movie durations, we repeated all ISC analyses in two replication datasets: HCP (Van Essen et al., 2013) and Emo-FilM (Morgenroth et al., 2025). In both datasets, we also conducted IS-RSA using Big Five personality traits as a well-established behavioral construct. These analyses constitute conceptual rather than direct replications because the behavioral measure used in the discovery dataset was not available in the replication datasets.

#### 3.5.1 ISC varies across movies in HCP and Emo-FilM datasets

We first computed ISC across the cortex for each movie. As shown in Supplementary Figures S12 and S13, the overall spatial distribution of ISC evoked by each of the 28 movie clips and films in the 2 replication datasets largely resembled patterns reported in previous movie-fMRI studies and those observed in the discovery dataset. One notable exception was Movie #11 in the HCP dataset, which showed relatively low ISC outside visual cortex (X. Li et al., 2025).

Next, we tested whether whole-brain ISC varied between movies. In both replication datasets, the ANOVA revealed a significant main effect of Movie: HCP, *F*(13, 1144) = 17.47, *p* < 0.001, η^2^_G_ = 0.13; Emo-FilM, *F*(13, 169) = 2.59, *p* = 0.003, η^2^_G_ = 0.089. We then examined the spatial distribution of ISC variability across the cortex. For each of the 210 cortical parcels defined by the Brainnetome atlas, we conducted a repeated-measures ANOVA on ISC values with Movie as the within-subject factor. In both replication datasets, we found statistically significant between-movie ISC variability distributed across the cortex (FWE-adjusted *p* < 0.05). Parcel-wise statistical values quantifying between-movie variability are available online (https://dx.doi.org/10.17605/OSF.IO/S78WU). Figure 5A shows the spatial distribution of between-movie ISC variability in the HCP dataset, and Figure 5B shows the corresponding results for the Emo-FilM dataset.

**Fig. 5.**
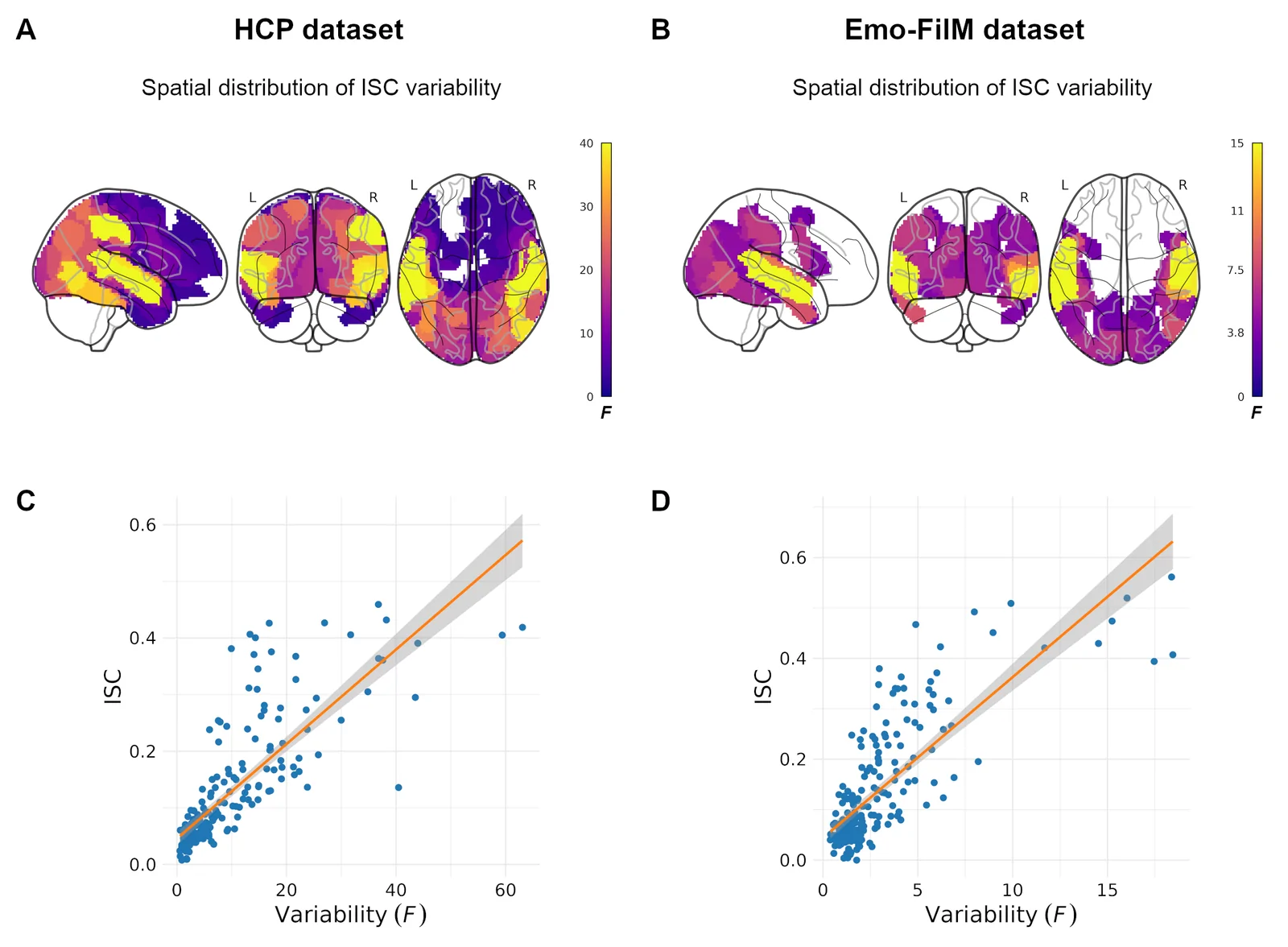
Variability in inter-subject correlations across movies in the two replication datasets (HCP and Emo-FilM). Fisher *z*-transformed ISC is shown for panels C and D. (A) Spatial distribution of statistically significant between-movie ISC variability across the brain in the HCP dataset (family-wise error-adjusted *p* < 0.05). (B) Spatial distribution of statistically significant between-movie ISC variability across the brain in the Emo-FilM dataset (family-wise error-adjusted *p* < 0.05). (C) Strong positive correlation between ISC level (y-axis) and between-movie variability (x-axis) across brain regions in the HCP dataset. Each point represents one cortical parcel. (D) Strong positive correlation between ISC level (y-axis) and between-movie variability (x-axis) across brain regions in the Emo-FilM dataset. Each point represents one cortical parcel.

For each replication dataset separately, we calculated the Pearson correlation between parcel-wise ISC level and between-movie ISC variability across the 210 cortical parcels. As shown in Figure 5C and D, ISC level was strongly positively correlated with between-movie variability: HCP, *r* = 0.80, *t*(208) = 19.16, *p* < 0.001, *R*^2^ = 0.64; Emo-FilM, *r* = 0.78, *t*(208) = 18.16, *p* < 0.001, *R*^2^ = 0.61. Together, these results show that our ISC findings were robust across independent datasets with live-action and animated movies of different durations.

#### 3.5.2 IS-RSA results for a well-validated construct vary across movies

We used IS-RSA to identify brain regions in which similarity between subjects in personality traits, measured with established questionnaires (John et al., 1991; McCrae & Costa, 2004), was associated with ISC during movie watching. In other words, we tested whether subjects with more similar personality profiles showed stronger neural synchronization in specific brain regions. These analyses conceptually extend prior work linking ISC to personality, in which associations were assessed across concatenated movie clips and, therefore, averaged across movie-specific effects (Finn et al., 2020; Matz et al., 2022).

For each replication dataset and movie separately, we performed regression-based IS-RSA using linear mixed-effects models. As shown in Supplementary Figures S14 and S15, IS-RSA revealed associations between personality similarity and ISC in distinct brain regions for each movie. In the HCP dataset, significant positive associations between personality similarity and ISC were observed for every movie (Supplementary Table S4). The spatial extent ranged from a single right posterior superior temporal sulcus parcel for Movie #2 to more than 30 brain regions for Movies #5 and #6. The strongest commonalities across movies were observed in the left middle temporal gyrus (Movies #3, #4, #5, #6, and #7) and right posterior superior temporal sulcus (Movies #2, #3, #5, #6, and #14), each significant for five movies. Nevertheless, most significant parcels were specific for a single movie, and the spatial extent of associations varied markedly across movies. In the Emo-FilM dataset, significant positive associations between personality similarity and ISC were observed for 13 of the 14 movies; no significant associations were observed for Movie #2 (Supplementary Table S5). The number of significant parcels ranged from 2 for Movies #8 and #10 to 19 for Movie #12. The clearest commonality was an association in the left inferior parietal lobule, which was significant for seven movies (Movies #1, #3, #4, #6, #10, #12, and #14); all other parcels recurred in no more than three movies, again indicating considerable differences in the extent and spatial distribution of associations across movies. Full parcel-wise results for all movies are available online (https://dx.doi.org/10.17605/OSF.IO/S78WU). Correlations between movie-specific, unthresholded IS-RSA *t*-maps were low in both replication datasets: HCP, mean *r* = 0.05 ± 0.13; Emo-FilM, mean *r* = 0.10 ± 0.15. This indicates limited consistency in the spatial pattern of ISC–behavior associations across movies, independent of the applied statistical threshold. Together, the IS-RSA results from the replication datasets show that movie-specific ISC–behavior associations are replicable across datasets and extend to a well-established behavioral construct.

## 4 Discussion

Here, we quantified the extent of ISC variability between different movies and assessed its consequences for ISC–behavior associations during movie watching. Across three independent datasets, our analyses revealed considerable between-movie variability in whole-brain ISC, and we found statistically significant variability distributed across the cortex. Using IS-RSA, we showed that associations between ISC and two distinct behavioral constructs had movie-specific spatial distributions.

### 4.1 Implications for interpreting ISC–behavior associations

ISC–behavior associations showed limited consistency across movies, with little overlap among statistically significant associations and low correlations between unthresholded movie-specific IS-RSA maps. This has two important implications for interpreting ISC-based studies. First, findings obtained from a single movie should be interpreted in the context of that particular movie. This constrains the generalizability of single-movie findings: without testing additional movies, there is limited basis for assuming that the same or a similar spatial pattern would emerge with another movie that could have been used to address the same research question. Second, our findings have implications for studies that concatenate several movies or average effects across them. Such approaches provide an aggregate estimate of the association across the included movies but can obscure marked heterogeneity between individual movies. A significant association obtained after concatenation or averaging may, therefore, be driven by only a subset of movies, while being weak or absent for others. Conversely, movie-specific associations that occur in different brain regions may be attenuated or obscured when aggregated. Accordingly, concatenated or averaged ISC-based results should be interpreted as effects specific to the sampled set of movies. Generalizability across movies is, therefore, best established empirically by showing that ISC–behavior associations recur across multiple movies, rather than inferred from findings based on a single movie or an aggregated movie set.

### 4.2 ISC strength and sensitivity to movie differences

Across all three datasets, brain regions with stronger ISC showed greater between-movie variability. We interpret this primarily as reflecting the stimulus-driven nature of ISC: regions with stronger synchronization are more strongly driven by stimulus features that elicit similar responses across subjects and may, therefore, be more sensitive to differences in movie content. At the same time, stronger ISC also provides a larger observable range in which between-movie differences can emerge. This statistical explanation is closely related to the neuroscientific one: regions with little stimulus-locked signal offer limited scope for systematic movie effects, whereas strongly synchronized regions offer more. Thus, the positive association likely reflects a close coupling between the strength of stimulus-locked synchronization and its sensitivity to the specific movie.

### 4.3 Limitations and future directions

A limitation of our study is that all three datasets used relatively short films or movie clips. It, therefore, remains to be determined whether ISC patterns and ISC–behavior associations show comparable movie specificity for feature-length movies, for example, from the naturalistic neuroimaging database (Aliko et al., 2020).

In the discovery dataset (CABB), we tested an IS-RSA model that included movie as a random effect. This analysis revealed consistent associations in a small number of brain regions, but most effects remained movie specific. However, because our random-effects model included only eight movies, variance components for the movie effect were estimated from a small number of movies, limiting the precision of these estimates and the strength of conclusions about generalization beyond the sampled movies (Judd et al., 2012).

An important future direction concerns the role of functional alignment. Our analyses were based on anatomically aligned, parcel-averaged time series and, therefore, did not account for individual differences in fine-grained functional topographies. Methods such as hyperalignment or the shared-response model address this issue by estimating subject-specific transformations into a common functional space and increase sensitivity to shared responses across individuals (Chen et al., 2015; Guntupalli et al., 2016; Haxby et al., 2011, 2020). Future studies analyzing voxel- or vertex-level responses to feature-length movies could use such data to estimate robust functional-alignment transformations. Alternatively, datasets with several sufficiently long movie clips could use leave-one-movie-out cross-validation, training the alignment on all but one movie clip and testing whether movie-specific ISC and ISC–behavior associations persist for the held-out clip.

Future work could also compare variability in responses to movies with variability across cognitive tasks that target similar underlying processes (Andermane et al., 2019; Cai et al., 2024; Fedorenko et al., 2013). Such comparisons may help contextualize movie-induced variability relative to more traditional task-based paradigms. Making such a comparison meaningful would require identifying the specific perceptual, cognitive, or affective processes that a given set of movies is likely to engage.

### 4.4 Conclusion

Across three independent datasets, we quantified how ISC patterns vary across movies and showed that ISC–behavior associations had distinct spatial distributions, with little overlap between movies. These findings suggest that ISC–behavior associations can be strongly movie specific, constraining the generalizability of findings based on a single movie and the interpretation of effects aggregated across multiple movies. Together, these findings highlight the importance of considering movie specificity when interpreting ISC–behavior associations.

## Supplementary Material

Supplementary Material

## Data Availability

Raw data from the CABB dataset are available in the Radboud Data Repository, as described in the accompanying data descriptor (https://doi.org/10.1016/j.neuroimage.2022.119734). The HCP data are publicly available and can be accessed through the HCP website (https://db.humanconnectome.org/). The Emo-FilM dataset is publicly available on OpenNeuro (https://openneuro.org/datasets/ds004892). The *Python* and *R* code used for the analyses is available on Github (https://github.com/simonleipold/movie_variability) and archived on Zenodo (https://doi.org/10.5281/zenodo.13348505).
